# Testosterone and socioeconomic position: Mendelian randomization in 306,248 men and women in UK Biobank

**DOI:** 10.1126/sciadv.abf8257

**Published:** 2021-07-28

**Authors:** Sean Harrison, Neil M. Davies, Laura D. Howe, Amanda Hughes

**Affiliations:** 1 MRC Integrative Epidemiology Unit, University of Bristol, Bristol, UK.; 2 Population Health Sciences, Bristol Medical School, University of Bristol, Bristol, UK.; 3 K.G. Jebsen Center for Genetic Epidemiology, Department of Public Health and Nursing, Norwegian University of Science and Technology, Norway.

## Abstract

Men with more advantaged socioeconomic position (SEP) have been observed to have higher levels of testosterone. It is unclear whether these associations arise because testosterone has a causal impact on SEP. In 306,248 participants of UK Biobank, we performed sex-stratified genome-wide association analysis to identify genetic variants associated with testosterone. Using the identified variants, we performed Mendelian randomization analysis of the influence of testosterone on socioeconomic position, including income, employment status, neighborhood-level deprivation, and educational qualifications; on health, including self-rated health and body mass index; and on risk-taking behavior. We found little evidence that testosterone affected socioeconomic position, health, or risk-taking. Our results therefore suggest that it is unlikely that testosterone meaningfully affects these outcomes in men or women. Differences between Mendelian randomization and multivariable-adjusted estimates suggest that previously reported associations with socioeconomic position and health may be due to residual confounding or reverse causation.

## Teaser

Mendelian randomization suggests circulating testosterone does not meaningfully affect men’s socioeconomic position.

## INTRODUCTION

Testosterone has long been of interest in the study of human behavior (*1*). For human and nonhuman primates, testosterone is thought to play a role in advancing and maintaining status compared to competitors (*2*, *3*). In experimental settings, it has been shown to promote either aggressive or prosocial behavior depending on the context (*2*) and to influence economic decision-making, particularly financial risk-taking (*4*, *5*). Outside the laboratory, there are reasons to believe that these same processes could, over longer time scales, affect people’s social and economic circumstances. Work in male occupational samples points to a positive relationship of testosterone with aspects of socioeconomic position (SEP). Among male executives, circulating testosterone has been linked with number of subordinates (*6*) and among male financial traders, with daily profits (*7*). Other research has suggested that the in utero exposure to testosterone increases adult earnings for men (*8*) but a possible detrimental association for women (*9*, *10*). However, the effects of testosterone exposure in utero and in adulthood may be very different since the former may have an organizational (permanent) influence on an organism and a transitory influence on its functioning (*11*), and an individual’s exposure to testosterone in utero and in adulthood may differ (*12*). Observational studies of adults where testosterone and outcomes are measured together meanwhile tell us little about causality. Circulating testosterone may causally affect earnings, employment status, or other aspects of a person’s SEP through influence on behavior. One hypothesized pathway involves a positive effect of testosterone on risk tolerance. This may bring financial rewards in the course of a career, at least in some professions (*7*), or influence a person’s choice of occupation (*13*). Circulating testosterone has also been linked to a greater likelihood of self-employment, a financially “riskier” strategy than standard employment (*14*). Second, insofar as testosterone promotes competitive or antagonistic behavior, it may increase willingness to engage in, or effectiveness in, wage bargaining. This has been proposed as a mechanism linking personality to socioeconomic outcomes within and between genders (*15*). With the exception of occupational choice, these processes may operate throughout a person’s career, potentially leading to accumulation of differences in SEP throughout adulthood. One aspect of SEP to which this would not apply is educational attainment (e.g., having a university degree). Since this is usually determined by early adulthood, it may be directly influenced by effects of testosterone on brain development (*11*). Thus, an association of midlife circulating testosterone with educational attainment may not indicate a causal effect of later-life levels of testosterone on SEP but instead reflect influences of early-life testosterone levels, which are affected by similar genetic variation. Similarly, because educational attainment strongly influences later income, associations of midlife SEP with circulating testosterone may partly reflect an impact of earlier testosterone on educational attainment. At the same time, there are reasons to believe that SEP may influence testosterone levels (i.e., reverse causation). Circulating testosterone is affected by nongenetic influences, including age (it declines across most of the adult lifespan for men and women) (*16*) and time of day (it is higher in the morning) (*17*). Chronic stress can lower testosterone levels by affecting both production and secretion (*18*), suggesting that psychosocial stress associated with socioeconomic adversity could influence testosterone alongside other aspects of health (*19*). SEP could also influence testosterone via obesity, which, in men, lowers circulating testosterone (*20*), and is associated with disadvantage in high-income countries. Because smoking is more common in less-advantaged groups but may raise testosterone, smoking could reduce rather than contribute to social differences in testosterone in men (*21*). Socioeconomic influences on testosterone could also be mediated by self-perception of social status, a dimension of SEP whose influences on health may be distinct from income (*22*). In competitive situations—both experimental settings and sports matches (*23*)—testosterone has been found to change depending on outcome, rising in the winner compared to the loser (*1*, *24*). Last, associations of circulating testosterone with SEP could be explained by the relationship of testosterone with other aspects of health. For men, observational studies looking at naturally occurring variation in testosterone have shown that higher testosterone is not only linked with better health, including lower risk of cardiovascular outcomes (*25*) and of type 2 diabetes (*26*), but also increased risk of prostate cancer (*27*). In women, higher testosterone has been associated with poorer metabolic health, including cardiovascular outcomes (*28*) and type 2 diabetes (*26*). Higher levels of testosterone and other androgens is also a key diagnostic feature of polycystic ovary syndrome (*29*). It is unclear how much these associations reflect the impact of testosterone on health as opposed to the impact of health on testosterone. Recent genetic evidence about its impact on cardiovascular health and associated risk factors (*30*, *31*) does, however, indicate that testosterone is likely to have some causal impacts on health. This includes blood pressure, lipids (*31*), and risk of type 2 diabetes (*30*). One recent Mendelian randomization (MR) study suggested a beneficial influence on some aspects of health (bone mineral density and body fat) but a detrimental impact on others (high-density lipoprotein cholesterol, hypertension, and prostate cancer risk) (*32*). Since health is known to influence diverse aspects of SEP (*33*), testosterone may therefore also affect SEP via health.

This evidence is, however, largely circumstantial, and despite plausible mechanisms, the relationship between testosterone and SEP is poorly understood. One issue is that most work has been experimental or involved specific occupational samples. Understanding associations more widely requires investigation in general population samples, since biological correlates of success for financial traders and sports players may not extend to the rest of the population. Second, both experimental and observational work has, to date, focused almost exclusively on men, and consequently, little is known about how testosterone relates to socioeconomic circumstances in women. Third, few studies have used causal inference methods to infer the effects of testosterone. Observational studies that do not are likely biased by confounding and reverse causation. This occurs even when a list of known confounders is included as covariates, since there may be substantial error in the measurement of those covariates, leading to “residual” confounding. A U.K. study from 2015 found social differences in men’s circulating testosterone at age 60 to 64, with lower testosterone for men with lower income and fewer educational qualifications (*34*), but could not determine whether this relationship was causal. A 2018 study in British men (*17*) applied MR, a genetic causal inference approach, to investigate the influence of circulating testosterone on SEP. It found suggestive evidence of a positive influence on earnings and probability of being in work, but the sample size (*N* = 3663) was insufficient to draw meaningful conclusions.

We investigated the impact of circulating testosterone on social and economic outcomes in 306,248 men and women from the UK Biobank. We investigated whether the associations of SEP and testosterone were likely to be causal using MR. MR uses genetic variants associated with an exposure of interest (usually single nucleotide polymorphisms or SNPs) as instrumental variables for the exposure, in this case, testosterone. Since SNPs are assigned at conception, associations with SNPs cannot be due to reverse causation or classical confounding of the exposure and the outcome (*35*). Multiple SNPs associated with an exposure can be combined into a polygenic score (PGS) representing overall genetic predisposition for the exposure. SNPs for the exposure are usually taken from an existing genome-wide association study (GWAS) based on a separate population to that in which the outcome is measured, because using the same population can result in bias (*36*). However, existing GWAS of testosterone not involving the UK Biobank was based on small samples (*37*, *38*) and identified few SNPs. We therefore conducted a GWAS of testosterone in the UK Biobank. We used a split-sample approach to delineate two independent samples (detailed in Materials and Methods), thus avoiding bias caused by overlap of the GWAS sample with the sample in which the outcome is measured (*36*). We examine the impact of testosterone on the following outcomes: household income, having a university degree, employment status, having a skilled job, neighborhood-level deprivation [Townsend Deprivation Index (TDI)] at recruitment, home ownership, and partnership/cohabitation status. We also examine associations with factors proposed as mediators of testosterone-SEP associations: overall health, adiposity [body mass index (BMI)], and risk-taking behavior. As a negative control, we examine the impact of testosterone with place of birth within the United Kingdom (North and East coordinates), which should not be predicted by testosterone. We also consider the reverse-causal pathway, an influence of SEP on testosterone, using SNPs previously associated in an independent sample with years of schooling to instrument educational attainment (*39*). Building on previous work restricted to men, we examine all relationships for men and women separately. Associations are expressed in terms of an SD change in testosterone, comparable to changes observed following interventions. For example, the SD of 3.69 nM for men’s total testosterone is similar to increases reported with moderate diet–induced weight loss among men with obesity (*20*) and with testosterone therapy in hypogonadal men (*40*). Our previous work (*33*) has suggested that diverse aspects of health may causally influence SEP. For instance, in MR models, a 5 kg/m^2^ increase in BMI was associated with greater probability of being out of work [absolute percentage change (APC): 1.4%; confidence interval (CI): 0.4 to 2.5%], lower probability of having a skilled job (−2.3%; CI: −3.5 to −1.0%), and lower probability of having a university education (−2.9%; CI: −4.4 to −1.5%). Effects per SD change in testosterone could plausibly fall in a similar range.

## RESULTS

Summary demographics are presented in Table 1. Briefly, we analyzed 148,248 men, 36,203 premenopausal women, and 104,632 postmenopausal women. The mean bioavailable testosterone level was 5.21 nM in men (SD, 1.54 nM), 0.37 nM in premenopausal women (SD, 0.25 nM), and 0.36 nM in postmenopausal women (SD, 0.28 nM). The PGS for bioavailable testosterone in each split had *R*^2^ values of 3.3% (from 41 SNPs) and 3.5% (from 40 SNPs) for men, 0.7% (from 6 SNPs) and 0.7% (from 7 SNPs) for premenopausal women, and 0.04% (from 76 SNPs) and 0.4% (from 5 SNPs) for postmenopausal women; table S1 details the PGS for all exposures, and table S2 details all the GWAS-significant SNPs identified in the GWAS.

**Table 1:** Summary demographics of the sample population.

**Variable**	**Men** **(*N* = 148,248)**	***N***	**Women** **(*N* = 158,000)**	***N***	**Premenopausal** **women** **(*N* = 36,203)**	***N***	**Postmenopausal** **women** **(*N* = 104,632)**	***N***
Age at recruitment, years [means (SD)]	57.1 (8.10)	148,248	56.7 (7.94)	158,000	46.4 (4.24)	36,203	60.5 (5.38)	104,632
Body mass index, kg/m^2^ [means (SD)]	27.8 (4.21)	147,766	27.1 (5.16)	157,516	26.4 (5.24)	36,131	27.2 (5.04)	104,274
**Exposures**								
Bioavailable testosterone, nM [means (SD)]	5.21 (1.54)	135,020	0.36 (0.27)	121,878	0.37 (0.25)	29,987	0.36 (0.28)	78,887
Free testosterone, nM [means (SD)]	0.21 (0.06)	135,020	0.01 (0.01)	121,878	0.02 (0.01)	29,987	0.01 (0.01)	78,887
Total testosterone, nM [means (SD)]	11.96 (3.69)	146,869	1.12 (0.64)	134,701	1.22 (0.60)	33,379	1.08 (0.66)	86,956
Albumin, g/liter [means (SD)]	45.53 (2.61)	136,952	45.02 (2.58)	145,875	44.91 (2.59)	33,043	45.05 (2.56)	96,955
SHBG, nM [means (SD)]	39.93 (16.74)	135,891	61.39 (29.55)	144,423	68.07 (32.31)	32,607	59.45 (28.03)	96,096
Outcomes*								
Annual pretax household income [means (SD)]	£50,570 (£35,328)	106,074	£50,817 (£34,822)	77,798	£55,580 (£36,362)	32,800	£46,863 (£33,021)	35,420
<£18,000 [*N* (%)]	16,417 (15.48)	16,417	11,265 (14.48)	11,265	3680 (11.22)	3680	6060 (17.11)	6060
£18,000 to £30,999 [*N* (%)]	23,164 (21.84)	23,164	16,621 (21.36)	16,621	5887 (17.95)	5887	8618 (24.33)	8618
£31,000 to £51,999 [*N* (%)]	30,904 (29.13)	30,904	23,631 (30.37)	23,631	10,219 (31.16)	10,219	10,541 (29.76)	10,541
£52,000 to £100,000 [*N* (%)]	27,979 (26.38)	27,979	20,886 (26.85)	20,886	10,170 (31.01)	10,170	8240 (23.26)	8240
>£100,000 [*N* (%)]	7610 (7.17)	7610	5395 (6.93)	5395	2844 (8.67)	2844	1961 (5.54)	1961
Townsend deprivation index (TDI) [means (SD)]	−1.5 (2.98)	148,072	−1.6 (2.87)	157,810	−1.5 (2.93)	36,147	−1.7 (2.84)	104,510
Employed [*N* (%)]	83,954 (72.11)	83,954	70,064 (79.58)	70,064	30,994 (86.47)	30,994	30,260 (73.55)	30,260
Unemployed [*N* (%)]	2857 (2.45)	2857	1180 (1.34)	1180	430 (1.20)	430	563 (1.37)	563
Out of labor force [*N* (%)]	5453 (4.68)	5453	3515 (3.99)	3515	778 (2.17)	778	2110 (5.13)	2110
Retired [*N* (%)]	22,012 (18.91)	22,012	5,611 (6.37)	5611	325 (0.91)	325	4745 (11.53)	4745
Looking after home/family [*N* (%)]	700 (0.60)	700	6037 (6.86)	6037	2715 (7.57)	2715	2607 (6.34)	2607
Skilled job [*N* (%)]	84,620 (84.22)	100,480	80,048 (81.20)	98,578	25,981 (83.34)	31,176	45,225 (80.31)	56,311
Degree level education [*N* (%)]	49,432 (40.52)	121,983	48,003 (37.02)	129,672	14,763 (42.72)	34,558	28,857 (35.54)	81,188
Own accommodation lived in [*N* (%)]	132,897 (90.81)	146,352	143,885 (92.12)	156,188	32,589 (90.95)	35,831	96,047 (92.86)	103,430
Self-reported risk-taker [*N* (%)]	48,522 (33.76)	143,716	27,315 (17.94)	152,238	7648 (21.94)	34,856	16,611 (16.45)	100,967
Self-reported poor/fair health [*N* (%)]	40,166 (27.19)	147,697	34,070 (21.64)	157,470	6569 (18.19)	36,105	22,566 (21.64)	104,279
Cohabiting [*N* (%)]	115,206 (78.12)	147,476	112,559 (71.59)	157,220	26,395 (73.07)	36,123	73,962 (71.09)	104,040
Current smoker [*N* (%)]	17,268 (11.65)	148,184	13,171 (8.34)	157,906	3468 (9.58)	36,188	8017 (7.67)	104,563
Lifetime smoking index [Median (IQR)]	0.0 (0.0 to 0.7)	147,722	0.0 (0.0 to 0.2)	157,473	0.0 (0.0 to 0.0)	36,135	0.0 (0.0 to 0.3)	104,254

*Household income and all employment variables were restricted to those younger than retirement age at the time of recruitment, i.e., under 60 years for women and under 65 years for men.

**Fig. 1:**
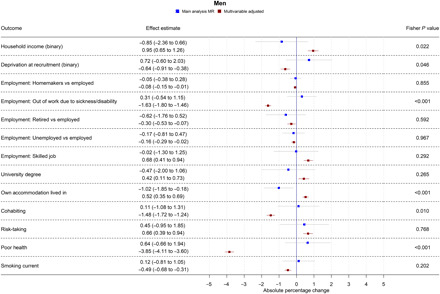
Forest plot showing the effect of bioavailable testosterone on all binary outcomes for men. Main analysis MR: Instrumental variable regression using PGS to instrument bioavailable testosterone, adjusted for age at baseline assessment, time of blood collection, UK Biobank recruitment center, and 40 genetic principal components. Multivariable-adjusted: Regression without genetic instruments, adjusted for age at baseline assessment, time of blood collection, UK Biobank recruitment center, and 40 genetic principal components. Note: Employment outcomes were restricted to men younger than 65 years at the time of recruitment.

**Fig. 2:**
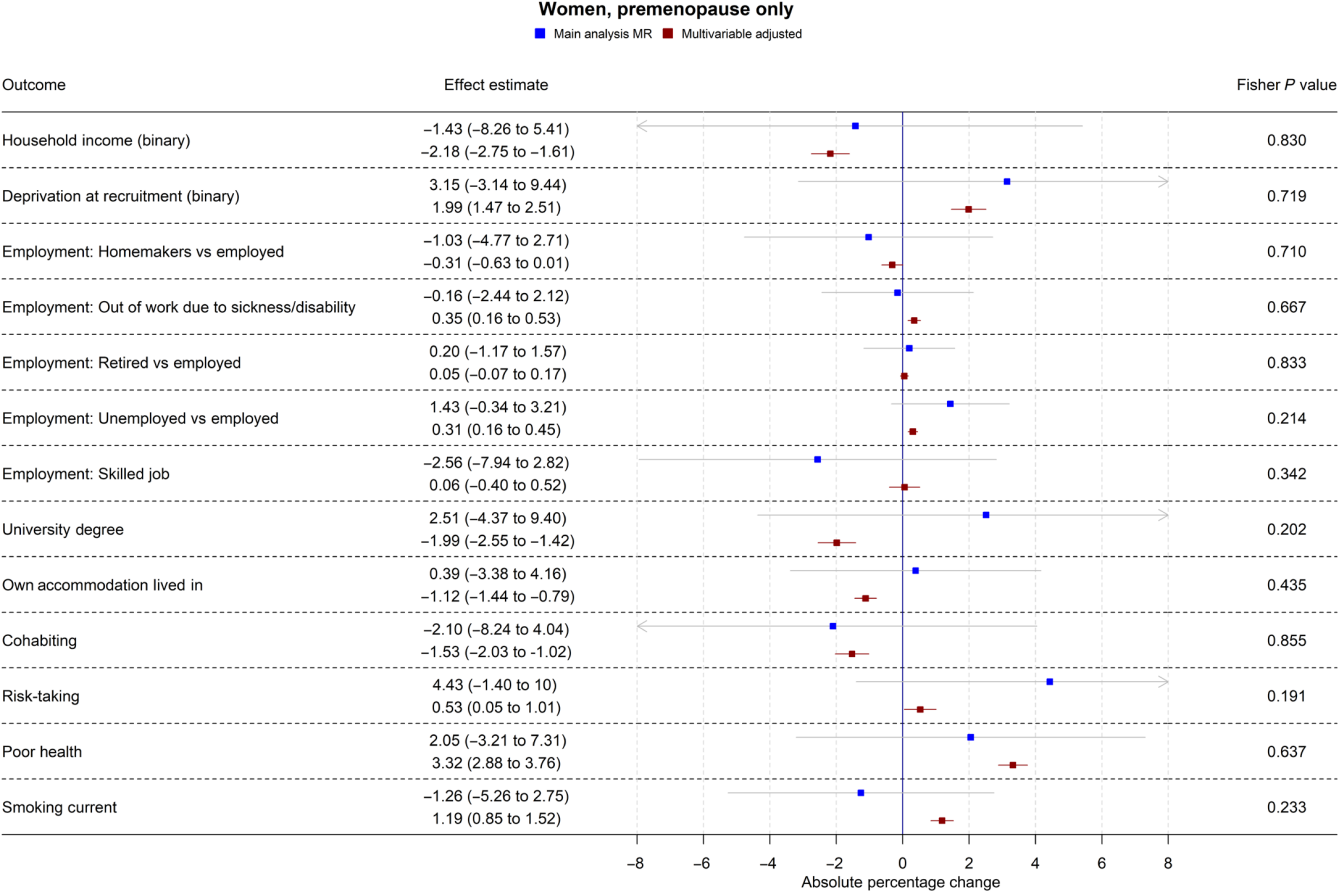
Forest plot showing the effect of bioavailable testosterone on all binary outcomes for premenopausal women. Main analysis MR: Instrumental variable regression using PGS to instrument bioavailable testosterone, adjusted for age at baseline assessment, time of blood collection, UK Biobank recruitment center, and 40 genetic principal components. Multivariable-adjusted: Regression without genetic instruments, adjusted for age at baseline assessment, time of blood collection, UK Biobank recruitment center, and 40 genetic principal components. Note: Employment outcomes were restricted to women younger than 60 years at the time of recruitment.

**Fig. 3:**
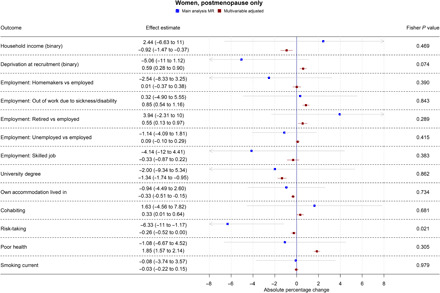
Forest plot showing the effect of bioavailable testosterone on all binary outcomes for postmenopausal women. Main analysis MR: Instrumental variable regression using PGS to instrument bioavailable testosterone, adjusted for age at baseline assessment, time of blood collection, UK Biobank recruitment center, and 40 genetic principal components. Multivariable-adjusted: Regression without genetic instruments, adjusted for age at baseline assessment, time of blood collection, UK Biobank recruitment center, and 40 genetic principal components. Note: Employment outcomes were restricted to women younger than 60 years at the time of recruitment.

**Fig. 4:**
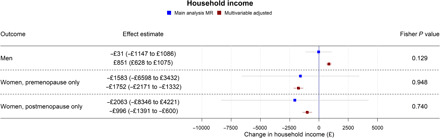
Forest plot showing the effect of bioavailable testosterone on household income for men, premenopausal women, and postmenopausal women. Main analysis MR: Instrumental variable regression using PGS to instrument bioavailable testosterone, adjusted for age at baseline assessment, time of blood collection, UK Biobank recruitment center, and 40 genetic principal components. Multivariable-adjusted: Regression without genetic instruments, adjusted for age at baseline assessment, time of blood collection, UK Biobank recruitment center, and 40 genetic principal components.

**Fig. 5:**
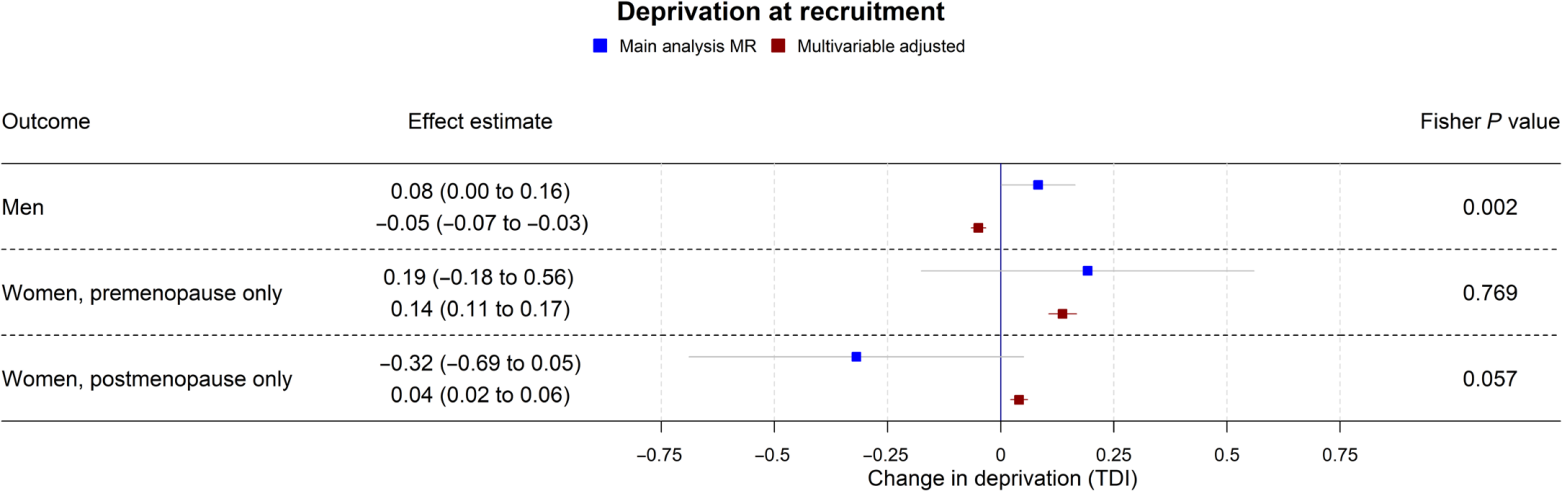
Forest plot showing the effect of bioavailable testosterone on neighborhood-level deprivation (TDI) for men, premenopausal women, and postmenopausal women. Main analysis MR: Instrumental variable regression using PGS to instrument bioavailable testosterone, adjusted for age at baseline assessment, time of blood collection, UK Biobank recruitment center, and 40 genetic principal components. Multivariable-adjusted: Regression without genetic instruments, adjusted for age at baseline assessment, time of blood collection, UK Biobank recruitment center, and 40 genetic principal components.

**Fig. 6:**
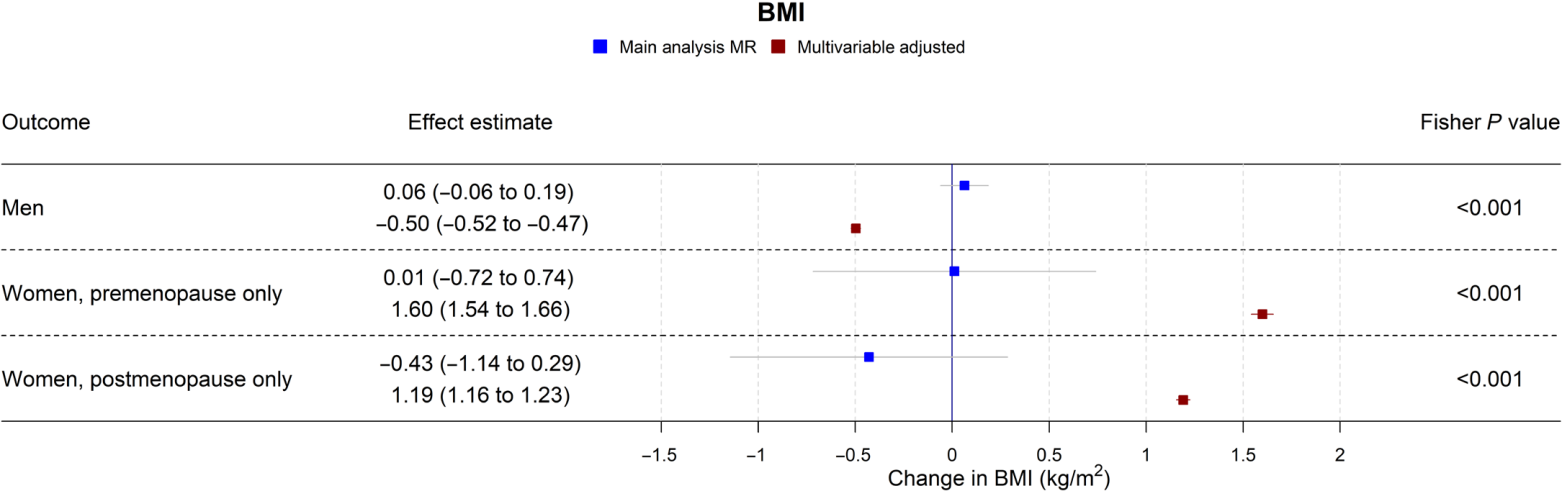
Forest plot showing the effect of bioavailable testosterone on BMI for men, premenopausal women, and postmenopausal women. Main analysis MR: Instrumental variable regression using PGS to instrument bioavailable testosterone, adjusted for age at baseline assessment, time of blood collection, UK Biobank recruitment center, and 40 genetic principal components. Multivariable-adjusted: Regression without genetic instruments, adjusted for age at baseline assessment, time of blood collection, UK Biobank recruitment center, and 40 genetic principal components.

**Fig. 7:**
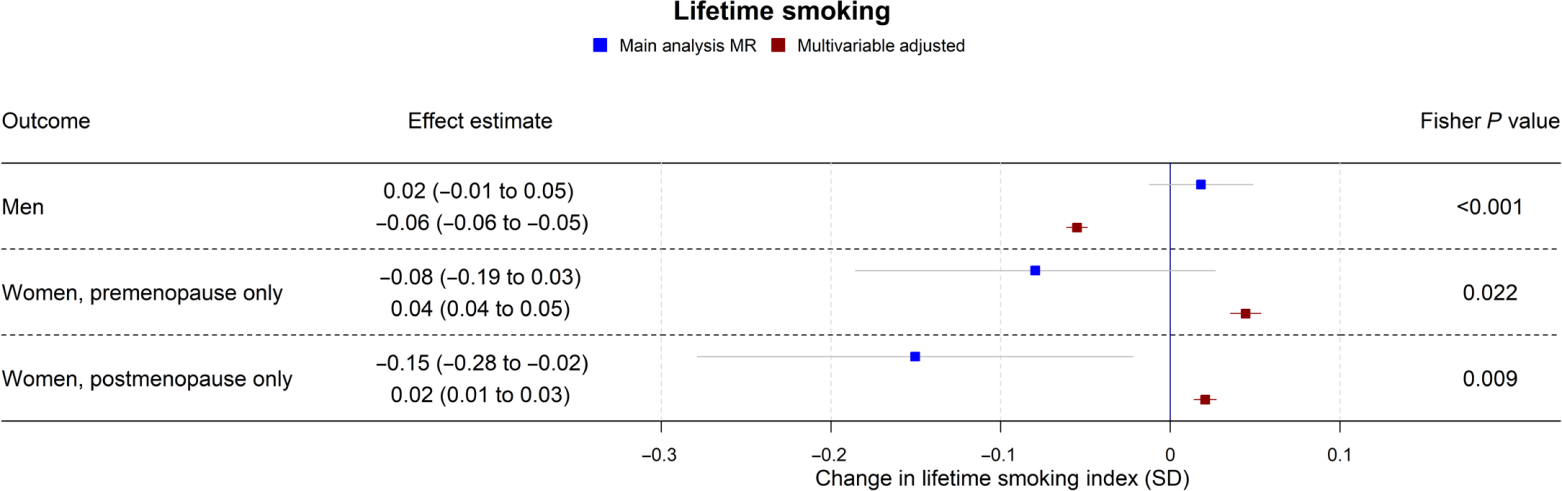
Forest plot showing the effect of bioavailable testosterone on lifetime-smoking index for men, premenopausal women, and postmenopausal women. Main analysis MR: Instrumental variable regression using PGS to instrument bioavailable testosterone, adjusted for age at baseline assessment, time of blood collection, UK Biobank recruitment center, and 40 genetic principal components. Multivariable-adjusted: Regression without genetic instruments, adjusted for age at baseline assessment, time of blood collection, UK Biobank recruitment center, and 40 genetic principal components.

Table S3 shows results from all the main and secondary analyses together. Forest plots showing the MR and multivariable-adjusted analysis results for binary outcomes are shown in Figs. 1 to 3. MR and multivariable-adjusted analysis results for continuous outcomes are shown in Figs. 4 to 7.

### Bioavailable testosterone in men

In multivariable-adjusted models, higher bioavailable testosterone in men was associated with more advantaged SEP (Figs. 1 and 4). For instance, a one-SD increase in bioavailable testosterone corresponded to £851 (95% CI: £628 to £1075) greater annual household income, greater likelihood of having a university degree (APC = 0.42%; 95% CI: 0.11 to 0.73%), and greater likelihood of holding a skilled job (APC = 0.68%; 95% CI: 0.41 to 0.94%). Bioavailable testosterone was also positively associated with health (Figs. 1 and 6). For instance, a one-SD increase in bioavailable testosterone corresponded to a 0.50-kg/m^2^ lower BMI (95% CI: 0.47 to 0.52) and lower probability of reporting poor health (APC = −3.85%; 95% CI: −4.11 to −3.60%). It was negatively associated with partnership (APC = −1.48%; CI: −1.72 to −1.24%), positively associated with self-assessed risk-taking (APC = 0.66%; 95% CI: −0.39 to 0.94%), and negatively associated with smoking, for example, corresponding to a decrease in the lifetime smoking index of 0.06 (95% CI: −0.06 to −0.05). This index captures initiation, duration, and heaviness, with a range in the sample of 0 to 5.81 and interquartile range (IQR) of 0 to 0.44 (more details are available in Materials and Methods).

In MR analyses (Figs. 1 and 4 to 7), effect sizes were smaller and CIs were larger. There was little evidence for causal effects of bioavailable testosterone on any outcome, with all 95% CIs crossing the null. For instance, a one-SD increase in men’s bioavailable testosterone corresponded to a difference in annual household income of −£31 (95% CI: −£1147 to £1086), an increase in BMI of 0.06 kg/m^2^ (95% CI: −0.06 to 0.19), and a difference in the lifetime smoking index of 0.02 (95% CI: −0.01 to 0.05). A one-SD increase in testosterone resulted in a −0.47 percentage points (95% CI: −2.00 to 1.06%) increase in the probability of having a university degree, −0.02% (95% CI: −1.30 to 1.25%) for likelihood of holding a skilled job, 0.64% (−0.66 to 1.94%) for reporting poor health, and 0.11% (−1.08 to 1.31%) for partnership. Although the CIs were wide, we were able exclude as very unlikely effect sizes beyond the upper and lower confidence limits of these estimates: for example, an impact of a one-SD increase in bioavailable testosterone on annual household income greater than £1086 or a decrease in BMI greater than 0.06 kg/m^2^. The MR estimate for owning one’s own accommodation was nominally significant and negative (−1.02; 95% CI: −1.85 to −0.18; *P* = 0.004) but should be considered in the context of the number of associations tested. Fisher tests (Figs. 1 and 4 to 7) showed that for several outcomes, the differences between the multivariable-adjusted and MR estimates were substantively different. This included for both multivariable-adjusted associations where the APC in the outcome exceeded 1: for being out of work due to sickness or disability (*P* = 1.02 × 10^−5^) and the chance of self-reporting poor health (*P* = 2.91 × 10^−11^). It was also the case for all continuous outcomes except household income: for neighborhood-level deprivation (*P* = 0.002), BMI (*P* = 1.56 × 10^−18^) and lifetime smoking (*P* = 4.12 × 10^−6^), and for the chance of owning accommodation (*P* = 4.28 × 10^−4^), where the MR estimate was larger than the multivariable-adjusted estimate [−1.02% (95% CI: −1.85 to −0.18%) versus 0.52% (95% CI: 0.35 to 0.69%)]. For all other outcomes, MR estimates were closer to the null than multivariable-adjusted estimates.

The PGS for bioavailable testosterone was not correlated with either the PGS for albumin or the PGS for sex hormone binding globulin (SHBG) (see table S4). There was little consistent evidence in both splits of heterogeneity between SNPs in the sensitivity MR analyses and little evidence of directional pleiotropy from MR-Egger regression (table S5). There was also a low risk of weak instrument bias for men; the *F* statistics in all analyses were above 1000.

### Bioavailable testosterone in premenopausal women

In multivariable-adjusted models, bioavailable testosterone among premenopausal women was generally associated with less advantaged SEP (Figs. 2 and 4 to 7). For instance, a one-SD increase in bioavailable testosterone corresponded to lower annual household income (−£1752; 95% CI: −£2171 to −£1332), lower probability of having a degree (APC = −1.99%; 95% CI: −2.55 to −1.42%), and lower probability of owning own accommodation (APC = −1.12%; 95% CI: −1.44 to −0.79%). It corresponded to a 1.60 kg/m^2^ (95% CI: 1.54 to 1.66) higher BMI (Fig. 6) and was positively associated with smoking, for instance, with an increase in the lifetime smoking index of 0.04 (95% CI: 0.04 to 0.05) (Fig. 7). As for men, bioavailable testosterone was negatively associated with partnership (APC per SD increase = −1.53%; 95% CI: −2.03 to −1.02%) and positively associated with self-assessed risk-taking (APC = 0.53%; 95% CI: 0.05 to 1.01%).

In MR analyses (Figs. 2 and 4 to 7), there was little evidence for any causal effects of bioavailable testosterone for premenopausal women. Differences per one-SD increase in bioavailable testosterone were −£1583 for annual household income (95% CI: −£6598 to −£3432), 0.01 kg/m^2^ for BMI (95% CI: −0.72 to 0.74 kg/m^2^), and −0.08 for the lifetime smoking index (95% CI: −0.19 to 0.03). For probability of having a degree, owning one’s own accommodation, and partnership, APCs were 2.51% (95% CI: −4.37 to 9.40%), 0.39% (95% CI: −3.38 to 4.16%), and −2.10% (95% CI: −8.24 to 4.04%), respectively. For self-assessed risk taking, the APC was 4.43% (95% CI: −1.40 to 10.26%). CIs were wider than for men, but a Fisher test indicated that for BMI, the MR estimate (beta = 0.01 kg/m^2^; 95% CI: −0.72 to 0.74 kg/m^2^) differed from the multivariable-adjusted estimate (*P* = 2.04 × 10^−5^). This was also the case for lifetime smoking (*P* = 0.007) and current smoking (*P* = 0.004). The PGS for bioavailable testosterone among premenopausal women was negatively correlated with the PGS for SHBG (*r* = −0.39), but other PGS were uncorrelated (absolute *r* < 0.005); see table S4. *F* statistics in all analyses were above 85, indicating that for premenopausal women, there was a low risk of weak instrument bias.

### Bioavailable testosterone in postmenopausal women

In postmenopausal women, bioavailable testosterone was negatively associated in multivariable-adjusted models with several measures of SEP and was associated with worse health outcomes (Figs. 3 and 4 to 7). A one-SD increase in bioavailable testosterone corresponded to £996 (95% CI: –£600 to £1391) lower annual household income, lower probability of owning own accommodation (APC = 0.33%; 95% CI: −0.51 to −0.15%), and higher TDI at recruitment 0.04 (0.02 to 0.06). It corresponded to a 1.19 kg/m^2^ (95% CI: 1.16 to 1.23) higher BMI, more lifetime smoking (beta = 0.02; 95% CI: 0.01 to 0.03), and greater likelihood of reporting poor health (APC = 1.85%; 95% CI: 1.57% to 2.14%). Unlike for men and for premenopausal women, bioavailable testosterone was positively associated with cohabitation (APC = 0.33%; 95% CI: 0.01 to 0.64%), and there was little evidence of an association with risk-taking (APC = −0.26%; 95% CI: −0.52 to 0.00%).

The MR estimates (Figs. 3 and 4 to 7) provided little evidence of a causal effect on most outcomes for postmenopausal women, but the estimates were imprecise. Differences per one-SD increase in bioavailable testosterone were as follows: for annual household income, −£2063 (−£8346 to £4221); for BMI, −0.43 kg/m^2^ (95% CI: −1.14 to 0.29 kg/m^2^); and for TDI at recruitment, −0.32 (95% CI: −0.69 to 0.05). For likelihood of owning one’s accommodation, of reporting poor health, and partnership, APCs were −0.94% (95% CI: −4.49 to 2.60%), −1.08% (−6.67 to 4.52%), and 1.63% (95% CI: −4.56 to 7.82%). For BMI, the Fisher test showed that the MR and multivariable-adjusted estimates were different (*P* = 9.19 × 10^−6^). A negative association for which the CI did not cross the null was seen with the lifetime smoking index −0.15 (95% CI: −0.28 to −0.02; *P* = 0.02) and self-assessed risk-taking −6.33% (95% CI: −11.49 to −1.17%; *P* = 0.02). In both cases, Fisher tests (*P* = 9.19 × 10^−6^, *P* = 0.02) gave evidence for a substantive difference between these estimates and the multivariable-adjusted estimates. However, MR estimates should be considered in the context of the number of associations tested. In postmenopausal women, the PGS for bioavailable testosterone was negatively correlated with PGS for SHBG (*r* = −0.25), but other PGS were uncorrelated (absolute *r* < 0.005); see table S4. *F* statistics for most analyses were above 10, except for some employment outcomes in split 1 [for being a homemaker (*F* = 6), being out of work due to sickness/disability (*F* = 9), having a skilled job (*F* = 8), and being unemployed (*F* = 6)]. Employment outcomes for postmenopausal women may therefore have a higher chance of weak instrument bias.

### Results of secondary analyses

Table S3 contains all results of secondary analyses together with main analyses. As in the main analyses, the CIs in MR analyses were wider than in multivariable-adjusted analyses. There was little evidence for effects of any exposure on any outcome for any group, with a few exceptions: There was some evidence that SHBG reduced BMI in premenopausal women (standardized beta = −0.35; 95% CI: −0.58 to −0.11; *P* = 0.004) and strong evidence from MR analyses that albumin reduced BMI in men (standardized beta = −0.39; 95% CI: −0.53 to −0.25; *P* = 4.8 × 10^−8^).

### Results of sensitivity analyses

Table S5 contains all results for sensitivity MR analyses, including pleiotropy-robust methods. There was little consistent evidence in both splits of heterogeneity between SNPs in the sensitivity MR analyses and little evidence of directional pleiotropy from MR-Egger regression. There was no strong evidence for an effect of bioavailable testosterone on any outcome in any sensitivity MR analysis (*P* > 0.01 in all combined analyses), although the effect estimates were imprecise. Similarly, there was no strong evidence of effects of albumin, total, and free testosterone on any main outcome in any sensitivity MR analysis (*P* > 0.01 in all combined split analyses). There was evidence of heterogeneity from MR-Egger regression between SHBG and some outcomes.

Table S7 contains results of negative control analyses. There was little evidence from MR models of associations of testosterone with place of birth (North and East coordinates within the United Kingdom). Tables S8 and S9 contain results for the impact of SEP (educational attainment) on testosterone. MR models using a PGS to instrument degree status (table S8) found was little evidence of effects of SEP on testosterone for men (e.g., for bioavailable testosterone, beta = 0.01; 95% CI: −0.14 to 0.16; *P* = 0.90). For women, there was evidence from MR estimates of positive effects of having a university degree on SHBG (for all women: beta = 0.40; 95% CI: 0.23 to 0.57; *P* = 2.7 × 10^−6^) and negative effects on bioavailable and free testosterone (for all women: beta = −0.35; 95% CI: −0.52 to −0.19; *P* = 2.9 × 10^−5^; beta = −0.35; 95% CI: −0.52 to −0.18; *P* = 3.5 × 10^−5^, respectively). Two-sample MR analyses (table S9) also found positive effects of education on women’s SHBG [for example, for all women: inverse-variance weighted (IVW) beta = 0.18; 95% CI: 0.08 to 0.29; *P* = 8.2 × 10^−4^] and negative effects of education on bioavailable and free testosterone (for all women: IVW beta = −0.15; 95% CI: −0.22 to −0.09; *P* = 8.5 × 10^−6^; IVW beta = −0.15; 95% CI: −0.22 to 0.09; *P* = 6.7 × 10^−6^, respectively). Two-sample MR analyses also suggested that education increases SHBG and decreases testosterone for men. For example, IVW estimates were respectively 0.32 (95% CI: 0.13 to 0.51; *P* = 8.2 × 10^−4^), −0.03 (95% CI: −0.04 to −0.01; *P* = 8.5 × 10^−6^), and −0.03 (95% CI: −0.04 to −0.02; *P* = 6.7 × 10^−6^) for SHBG, bioavailable, and free testosterone. There was little evidence from MR-Egger models of directional pleiotropy for any group.

## DISCUSSION

Consistent with existing observational work (*6*, *8*, *25*, *28*, *34*), multivariable-adjusted regression models found that bioavailable testosterone was associated with more advantaged SEP and better health among men but less advantaged SEP and worse health for women. In contrast, the MR estimates provided little evidence that circulating testosterone among adults had causal effects on SEP or health. Fisher tests provided substantial evidence of heterogeneity between the multivariable-adjusted and MR estimates. In men, the MR estimates were precise enough to suggest that even small effects of testosterone on SEP or health are unlikely. This contrasts with our previous work on BMI (*33*), where in MR models, a 5-kg/m^2^ increase in BMI was associated with SEP outcomes, including greater probability of being out of work (1.4%; CI: 0.4 to 2.5%) and lower probability of having a university degree (−2.9%; CI: −4.4 to −1.5%). In women, results of MR were less precise and are therefore consistent with a substantially larger range of effects. Since MR analyses are not affected by classical confounding or reverse causation of the exposure-outcome relationship, results suggest that many previously reported associations of testosterone with socioeconomic outcomes, self-rated health, and BMI among men are unlikely to be causal. The results of our MR estimates contrast with several studies of in utero testosterone exposure (*8*–*10*) supporting a causal impact on later socioeconomic outcomes. This may indicate a lasting influence specifically of prenatal testosterone exposure on these outcomes, consistent with the prenatal period being especially important for brain development (*11*). Unlike our study, a recent MR analysis (*30*) supported a causal impact of bioavailable testosterone on women’s BMI, although CIs for the two studies’ results overlapped. This may reflect different analytic decisions—for instance, that study conducted a GWAS in the whole UK Biobank population, with outcome information taken from a different study population entirely. Another MR study (*32*) supported an impact on body fat in men. Since in that study, the identification of genetic instruments and the MR were conducted in the same sample, without applying a split-sample approach, results may in part reflect bias because of sample overlap (*36*).

Risk-taking was in multivariable-adjusted regression models positively associated with testosterone for men and premenopausal women. The MR estimates provided little evidence of a positive relationship. This runs contrary to evidence from experimental settings (*4*, *5*, *41*) supporting a positive association of testosterone with risk-taking behavior. It could reflect lack of generalizability beyond experimental settings or the limitations of measuring risk-taking with a self-reported and subjective measure.

A previous MR study based on participants of the U.K. Household Longitudinal Survey (UKHLS) (*17*) found suggestive support for an impact of circulating testosterone on men’s earnings and probability of being employed, but the sample size was small (*N* = 3663) and estimates too imprecise to draw firm conclusions. UK Biobank collected information on household income but not individual earnings. It is possible that causal influence of testosterone on income relates to individual labor income specifically, although the current study found no evidence for an impact on men’s employment status either. One difference concerns representativeness of the study populations. UK Biobank participants are more socioeconomically advantaged and healthier than the general U.K. population. If they are also selected with respect to testosterone, null results could partly reflect bias due to selection, which can distort associations away from or toward the null (*42*). Comparing mean values of total testosterone corrected for time of day showed that men in the UK Biobank had slightly lower total testosterone than men in UKHLS but with substantial overlap [for example, 12.0 nM (SD, 3.7) for men aged 41 to 45, as opposed to around 15 nM in UKHLS]. Reference ranges for nationally representative populations are unavailable, so it is unclear which sample’s mean values are closer to those of all white British men. However, to explain our null findings, this bias due to selection would need to perfectly offset true effects. Another difference is that testosterone in the earlier study was instrumented with just three SNPs, the only variants at the time known to associate with testosterone. The most predictive of these was not available in the current study (nor were any SNPs that could be used as proxies). Since the current study used more SNPs, we were able to apply methods to investigate pleiotropy, and for this reason, the current study may be considered more robust.

Analyses using SNPs associated with educational attainment did suggest an impact of at least one aspect of SEP on testosterone for women. For men, results of different models were inconsistent, but for women, models using SNPs to instrument degree status and two-sample MR both suggested a positive impact of education on SHBG and a negative impact on bioavailable and free testosterone. These results are consistent with previously reported associations in women of sex hormones with adiposity (negative for SHBG and positive for testosterone) (*43*) and negative associations of education with adiposity. Together will null associations for the impact of testosterone on SEP, these results further suggest that multivariable-adjusted estimates and previously reported associations of testosterone and SEP may reflect influence of SEP on testosterone.

### Strengths and limitations

The foremost strength of this analysis is that MR analyses are not affected by classical kinds of confounding or reverse causation, which can affect other observational methods even when covariates are adjusted for (*35*). Given plausible mechanisms for an impact of SEP on testosterone, as well as the reverse, this is an important consideration. The UK Biobank is much larger than the studies previously used in this area—our analytic sample was approximately 84 times larger than that of the previous MR study on the topic (*17*). This allowed us to examine multiple health exposures and multiple socioeconomic and social outcomes with greater precision than previously possible. For some associations, there were marked differences between the MR and multivariable-adjusted estimates, which suggests that the multivariable-adjusted estimates may suffer from reverse causation or residual confounding. In addition, owing to the split-sample GWAS design, the SNPs contributing to the PGS were not biased by overlap between the GWAS and outcome sample populations (*36*), did not suffer from heterogeneity between the GWAS and analysis dataset, and all SNPs used reached genome-wide significance. Last, MR estimates for men are sufficiently precise to conclude that testosterone changes of the magnitude expected from clinical interventions are highly unlikely to result in clinically or economically relevant changes in SEP outcomes.

Risk-taking behavior was based on a subjective self-reported measure and may not adequately capture behavior relevant to testosterone. Recent evidence suggests that testosterone and cortisol jointly influence risk-taking (*5*), but measurements of cortisol were not available. Some of the proposed mechanisms linking testosterone to SEP, particularly the effects on educational attainment and on occupational choice, relate to periods of the lifespan, which we were unable to observe with our sample. Genetic influences of testosterone in early adulthood and midlife are likely to substantially overlap, but further investigation of these relationships is warranted in younger study populations, where relationships with circulating testosterone could differ. MR rests on assumptions that are difficult to test (*35*). Specification checks, including MR-Egger, weighted median, and weighted mode regression found little evidence of directional pleiotropy, but bias due to pleiotropy cannot be ruled out. The number of SNPs making up the PGS for bioavailable testosterone was low for premenopausal women, limiting statistical power. For postmenopausal women, the PGS included few SNPs in split 2 (SNPs = 5), and although more were identified in split 1 (SNPs = 76), they explained far less of the variance (*R*^2^ = 0.04% in split 1 and *R*^2^ = 0.4% in split 2). This is likely due to chance, as almost all the SNPs included in split 1 have small minor allele frequencies. The MR results for both premenopausal and postmenopausal women were therefore much less precise than for men and consequently consistent with a wider range of causal effects of testosterone. The PGS represents lifetime exposure to bioavailable testosterone, and effects at specific points in life cannot be explored with the methodology used here. Another important potential source of bias in MR analyses is family-level genetic effects, for example, the impact of parents’ genes on offspring via environmental pathways known as dynastic effects or genetic nurture. Recent evidence suggests that these processes are especially relevant to MR with socioeconomic outcomes, for instance, substantially distorting estimates of the causal impact of BMI on educational attainment (*44*). This may also occur for socioeconomic effects of testosterone. Methods robust to these biases exist but require data on large numbers of related individuals. These “within-family” MR methods have been applied in the UK Biobank using a stronger genetic instrument than was available here, but estimates were too imprecise to draw conclusions (*45*). For this research question, analysis would need to be further restricted to just same-sex sibling pairs. We have therefore not used within-family analyses here as power would be extremely limited. However, note that these phenomena tend to inflate estimated causal effects rather than push them toward the null. Our MR results were consistent with the null and, for men, relatively precise. Our findings are therefore unlikely to be due to ancestry, dynastic effects, or assortative mating.

Analyses were restricted to participants of white British ancestry, and results may not be generalizable to other groups. Participants of the UK Biobank tend to be wealthier and healthier than the country as a whole, and this nonrepresentativeness may bias estimates of effect sizes, including from genetic models (*42*). The UK Biobank is extremely limited in its measurements of early-life conditions and parental characteristics, and it was thus not possible to address these concerns by adjusting for early-life measures. Since bias due to selection can be toward or away from the null, we cannot rule out that our null results in part reflect influence of bias. However, to explain our null findings, the bias would need to perfectly offset true effects. This is unlikely. An additional source of potential bias is geographic structure in the UK Biobank genotype data. Frequencies of genetic variants differ between ancestral populations and hence between parts of the United Kingdom but so do environmental and cultural factors, which influence traits of interest independently of genetics. This may cause confounding that cannot be accounted for by adjusting for principal components. However, recent evidence suggests that while geographic structure may be present after controlling for principal components in the PGS for exposures associated with educational attainment (e.g. BMI and smoking), there was little evidence for geographic structure in other PGS (*46*), implying that bioavailable testosterone may be less subject to this bias. As with family-level effects, while these effects could explain a false positive, they are unlikely to explain our negative results. In conclusion, application of genetic causal inference methods in a large U.K. sample suggests that many previously reported associations of testosterone with socioeconomic outcomes, health, and risk-taking are unlikely to be causal.

## MATERIALS AND METHODS

### Experimental design

The objectives of this study were to investigate the causal effect of testosterone on SEP, health, and risk-taking behavior among adults and to assess whether previously reported associations are likely to reflect residual confounding or reverse causation. In a large survey of men, premenopausal, and postmenopausal women, we conducted split-sample GWAS to identify SNPs associated in each group with testosterone (bioavailable, free, and total), albumin, and SHBG. We then carried out split-sample MR, alongside multivariable-adjusted regression analyses, of the effect of these exposures on outcomes.

### Study sample

All analyses were based on preexisting data from the UK Biobank, a population-based health research resource consisting of 502,620 people recruited between 2006 and 2010 from 22 centers across the United Kingdom (*47*). Participants provided socioeconomic information and anthropometric measures via questionnaires and interviews at recruitment, and biomarkers were measured using blood tests. The study design, participants, and quality control methods have been described in detail previously (*47*, *48*). Participants provided electronically signed consent at baseline (*47*). The UK Biobank received ethics approval from the Research Ethics Committee (REC reference for the UK Biobank is 11/NW/0382).

Of the 502,620 participants with phenotypic data in the UK Biobank, 135 participants withdrew, leaving 502,506 participants. We then excluded those without a successful genotype (*N* = 14,241), those with sex-mismatch (derived by comparing genetic sex and reported sex), individuals with sex-chromosome aneuploidy, or outliers in heterozygosity and missing rates (*N* = 1,811), and participants related to a very large (>200) number of participants (*N* = 9), leaving 486,445 participants. We restricted the analyses to individuals of white British ancestry, as defined by participants who self-reported as “white British” and who had very similar ancestral backgrounds according to principal components analysis (*N* = 408,168), as described by Bycroft *et al.* (*48*). We excluded participants who reported at baseline that they were taking any drug that would influence their levels of testosterone (*N* = 410) or estrogen (*N* = 6134), including hormone replacement therapy (*N* = 7081) and the contraceptive pill (*N* = 2986); a full list of medications resulting in exclusion is available in table S6. A total of 15,857 participants were excluded for medications use, as some participants reported taking more than one testosterone- or oestrogen-altering drug. We also excluded 21,335 participants who had no measured levels of albumin, SHBG, or testosterone. A total of 107,162 pairs of related individuals had been previously identified (*48*). We applied an in-house algorithm to this list and preferentially removed the individuals related to the greatest number of other individuals until no related pairs remained. This resulted in the exclusion of 64,728 individuals from the MR analyses (although related participants were included in the split-sample GWAS as relatedness was accounted for). After exclusions, 370,976 (related) participants remained for the split-sample GWAS, and 306,248 unrelated participants remained for the MR analyses.

### Genetic data

The full data release contains the cohort of successfully genotyped samples (*n* = 488,377). A total of 49,979 individuals were genotyped using the U.K. BiLEVE array and 438,398 using the UK Biobank axiom array. Preimputation quality control, phasing, and imputation are described elsewhere (*48*). Briefly, before phasing, multiallelic SNPs or those with minor allele frequency (MAF) ≤ 1% were removed. Phasing of genotype data was performed using a modified version of the SHAPEIT2 algorithm. Genotype imputation to a reference set combining the UK10K haplotype and the Haplotype Reference Consortium (HRC) reference panels was performed using IMPUTE2 algorithms. The analyses presented here were restricted to autosomal variants within the HRC site list using a graded filtering with varying imputation quality for different allele frequency ranges. Therefore, rarer genetic variants are required to have a higher imputation INFO score (Info>0.3 for MAF >3%, Info>0.6 for MAF 1 to 3%, Info>0.8 for MAF 0.5 to 1%, and Info>0.9 for MAF 0.1 to 0.5%) with MAF and Info scores having been recalculated on an in house–derived “European” subset. Further information on the Medical Research Council Integrative Epidemiology Unit (MRC-IEU) quality control of the UK Biobank genetic data is available online (*49*).

### Testosterone

Testosterone circulates in several forms: unbound or “free”, loosely bound to albumin, or tightly bound to SHGB. Bioavailable testosterone refers to the first two categories, which are available for biological processes and hence more relevant to purported causal effects. The primary exposure in this analysis is bioavailable testosterone, but in additional analyses, we examined relationships with total and free testosterone. In the UK Biobank, testosterone and SHBG were measured by one-step competitive analysis on a Beckman Coulter Unicel Dxl 800 in nM, and albumin was measured by bromocresol green (BCG) analysis on a Beckman Coulter AU5800 in grams per liter. There were 23 participants with the minimum detectable limit of 0.35 nM testosterone; we coded these participants as having 0.35 nM testosterone. All participants had over the minimum detectable levels of albumin and SHBG.

We estimated free testosterone using the method detailed in Ho *et al.* (*50*), based on the work of Vermeulen *et al.* (*51*)

$$\text{FT}=\frac{T-0.5217A-S-1+\sqrt{{\left(0.5217A+1+S-T\right)}^{2}+4T\times (0.5217A+1)}}{2\times (0.5217A+1)}$$ (1)

where FT is free testosterone, *T* is total testosterone (nanomoles per liter), *S* is SHBG (nanomoles per liter), and *A* is albumin (grams per liter). Similarly, we estimated bioavailable testosterone as

BAT=FT+0.5217A×FT (2)

where BAT is bioavailable testosterone.

### Covariates

Age, sex, and the UK Biobank recruitment center were reported at the baseline assessment, and 40 genetic principal components [used to control for population stratification (*52*)] were derived by the UK Biobank. We classified participants as having had an oophorectomy if they answered yes to the question “Have you had BOTH ovaries removed?” and coded participants as having had menopause if they answered yes to “Have you had your menopause (periods stopped)?” or they had an oophorectomy. We coded the time of day of blood collection as the number of hours from midnight to the time of first blood collection.

### Outcomes

Continuous outcomes included household income, neighborhood-level deprivation, lifetime smoking behavior, and BMI. In the baseline questionnaire, participants reported annual household income before tax by choosing from wide categories. So that income could be treated as continuous measure, we used for each category the midpoint of the range (and for open-ended categories assigned a nominal value) as follows: <£18,000 = £15,000, £18,000 to £30,999 = £24,500, £31,000 to £51,999 = £41,500, £52,000 to £100,000 = £76,000, and >£100,000 = £150,000. In additional analyses, we dichotomized income as ≥£52,000 versus <£52,000. Neighborhood-level deprivation was measured using the TDI of current address, which, in our sample, had a range of −6.26 to 10.88 and an IQR of −3.75 to 0.03.

Lifetime smoking behavior was measured with an index developed in the UK Biobank, which captures initiation, heaviness, and duration. Details of the derivation are described elsewhere (*53*). BMI was calculated as participants’ weight in kilograms divided by their height in meters squared, based on measurements taken at the baseline assessment.

Binary outcomes included current employment status, job class, degree status, whether a participant lived in owner-occupied or rented accommodation, whether they were in a cohabiting partnership, self-reported risk-taking behavior, self-reported health, and currently smoking. Current employment status was coded as four separate outcomes:

1) Looking after home/family (homemaker) versus employed
2) Out of labor force (due to sickness/disability) versus employed
3) Retired versus employed
4) Unemployed versus employed

As in previous work (*33*), job class was coded as skilled versus unskilled. A skilled job was defined as the ones in the following categories:

1) Managers and senior officials
2) Professional occupations
3) Associate professional and technical occupations
4) Administrative and secretarial occupations
5) Skilled trade occupations.

Unskilled jobs were defined as the ones in the following categories:

1) Personal service occupations
2) Sales and customer service occupations
3) Process, plant, and machine operatives
4) Elementary occupations.

For risk-taking, participants were asked: “Would you describe yourself as someone who takes risks?” and could answer yes, no, do not know, or prefer not to say. From this, we constructed a binary yes/no variable, “no,” with small numbers of participants answering otherwise excluded. Self-reported overall health was dichotomized as good or excellent overall health versus poor or fair overall health. Degree status was coded as having a college or university degree versus not. We did not consider professional qualifications to be equivalent to degree-level education.

For household income and employment-related outcomes, we restricted the analysis to participants under the stage pension age at the time of recruitment (60 years for women and 65 years for men). Since these outcomes are closely linked with current labor market involvement, associations with testosterone would be expected to weaken considerably following retirement age. For all other outcomes, the full age range of the UK Biobank was used.

We created binary variables for household income and deprivation (as measured by TDI), which we analyzed along with the continuous measures. For household income, we compared those with a total household income above and below £52,000, i.e., upper two categories of household income versus bottom three categories. For deprivation, we split the participants into tertiles of TDI and compared the most deprived tertile with the remaining two tertiles.

### Statistical analysis

We analyzed men, premenopausal women, and postmenopausal women separately. Testosterone levels and genetic determinants differ substantially between men and women, and testosterone levels also differ between women who have and have not had an oophorectomy. We classified women who had reported having an oophorectomy as postmenopausal, and we excluded women for whom menopausal status was unknown from the main analyses.

As previous GWAS for bioavailable testosterone have been underpowered, we conducted new GWAS to identify genetic variants associated with bioavailable testosterone separately for men, premenopausal women, and postmenopausal women, retaining related participants. We used a split-sample approach, randomly splitting each of the three groups (men, premenopausal women, postmenopausal women) into two halves, or “splits” and conducting a GWAS in each split using the MRC-IEU UK Biobank GWAS pipeline (*54*). For men, premenopausal, and postmenopausal women, we then performed MR analyses separately in each of the two splits using the SNPs identified in the GWAS of the other. Conducting a split-sample analysis avoids bias from using overlapping GWAS and analytic samples (*36*) while maintaining statistical power. In all GWAS, we used BOLT-LMM (*55*) (which accounts for relatedness between participants and population structure), with age and 40 principal components as covariates to maximize power. In main analyses, for postmenopausal women, we also included having had an oophorectomy as a covariate (in secondary analyses, we ran MR models based on the GWAS without this covariate).

For men, premenopausal women, and postmenopausal women, we clumped the GWAS significant SNPs (*P* < 5 × 10^−8^) from each split’s GWAS with a clumping window of 10,000 kb and an *R*^2^ threshold of 0.001. We used the clumped SNPs to create a PGS for bioavailable testosterone in unrelated participants in the other split. We calculated the PGS as the sum of each individual’s testosterone-increasing alleles weighted by the regression coefficient from the GWAS.

Within each split, we used MR to estimate the causal effect of bioavailable testosterone on all outcomes, using the PGS as an instrumental variable, with age at baseline assessment (linear, squared, and cubed), time of blood collection (linear, squared, and cubed), UK Biobank recruitment center, and 40 genetic principal components as covariates. We used the ivreg2 package in Stata (version 15.1) with robust SEs and tested for weak instrument bias (using Kleibergen-Papp *F* statistics) to assess whether the PGS were sufficiently predictive of testosterone.

MR analyses estimate means and risk differences for continuous and binary outcomes, respectively, using additive structural mean models (*56*). Mean differences are interpreted as the average change in a participant’s outcome (e.g., household income) per unit increase in the exposure. Risk differences are interpreted as the absolute percentage point change in the proportion of participants with the outcome (e.g., unemployment) per unit increase in the exposure (as in a linear probability model).

For men, premenopausal women, and postmenopausal women separately, we used fixed-effect meta-analysis to combine the results from the two splits, giving a final estimate of the effect of bioavailable testosterone on each outcome. We express all results per SD increase in bioavailable testosterone, estimating the SD of bioavailable testosterone within men, premenopausal, and postmenopausal women separately.

To compare the MR results with associations from multivariable-adjusted analysis, within each split, we estimated multivariable-adjusted associations between bioavailable testosterone and all outcomes using linear regression, with age at baseline assessment (linear, squared, and cubed), UK Biobank recruitment center, time of day of blood collection (linear, squared, and cubed), and 40 genetic principal components as covariates, i.e., observational analyses without genetic instruments. We used linear probability models for binary outcomes rather than logistic regression models, to be comparable with the MR analyses. We also performed Hausman tests within each split to test whether estimates from MR and multivariable-adjusted analyses differed. As Hausman tests are only possible to conduct within each split, we used Fisher tests (*57*) to test whether estimates from the MR and multivariable-adjusted analyses differed after meta-analysis. All statistical tests were two-tailed.

### Sensitivity analyses

MR analyses are based on the assumption that SNPs, and therefore, PGSs are not pleiotropic, i.e., that they do not affect the outcome except through the exposure. We therefore conducted sensitivity MR analyses to test this assumption, including IVW, MR-Egger (which provides a test of certain forms of pleiotropy), weighted median, and weighted mode analyses within each split (*58*). These methods are able to detect pleiotropy under a range of conditions but also rely on assumptions (*59*). We also measured Cochran’s Q statistic from the IVW analyses (a measure of heterogeneity in the estimated effects on outcome using individual SNPs), an indicator of pleiotropy or problems with modeling assumptions (*59*). We combined the results of each analysis from each split using fixed effect meta-analysis, as in the main analysis.

We determined from these analyses (i) whether results of the sensitivity analysis were consistent with those from the main MR analysis, indicating robustness of the main results and (ii) whether there was evidence of pleiotropy from both the Egger regression constant term and Cochran’s Q statistic. We also inspected plots showing the results of the sensitivity MR analyses, which would indicate possible bias in the results of the main analysis.

As a negative control, we examined estimated associations from multivariable-adjusted and MR models of exposures with place of birth (North and East coordinates). These characteristics should not be associated with exposures beyond chance, and non-null associations could indicate possible bias.

To investigate possible reverse causality, we ran multivariable-adjusted and MR models for the impact on exposures of one aspect of SEP, namely, educational attainment. These analyses were based on SNPs previously associated with years of schooling in the largest GWAS, which did not include the UK Biobank (*39*). As for the main analyses, models were run separately for men, premenopausal, and postmenopausal women. First, we ran MR analyses using a PGS based on these SNPs to instrument degree status among participants in the analytic sample. For postmenopausal women, we conducted GWAS with and without adjustment for oophorectomy and ran MR models based on both sets of GWAS results. The results of these analyses are expressed as the SD change in the outcome for having a university degree versus not having a degree, with the SD estimated within men, premenopausal, and postmenopausal women separately. Second, we calculated associations of the individual SNPs from the years of schooling GWAS with testosterone, albumin, and SHBG in our analytic sample and used the associations to apply two-sample MR approaches: IVW, MR-Egger, weighted median, and weighted mode analyses (*58*). For postmenopausal women, we ran models based on SNP-exposure associations calculated with and without adjustment for oophorectomy. The results of these analyses are expressed as the SD change in the outcome for having an extra year of schooling, with the SD estimated within men, premenopausal, and postmenopausal women separately.

### Secondary analyses

We repeated the GWAS and all subsequent analyses for total testosterone, free testosterone, albumin, and SHBG. We also repeated the GWAS and all analyses for all women regardless of menopausal status [adjusting the GWAS and analyses for menopause (categorical: yes, no, and do not know) and oophorectomy], and, additionally, for postmenopausal women but without adjusting for oophorectomy.

As additional outcomes, we included participants above retirement age in household income and all employment variables, as well as equivalized household income, defined as household income divided by the number of people living in the same household, setting the maximum number in the household at 12.

### Patient and public involvement

This study was conducted using the UK Biobank (www.ukbiobank.ac.uk/about-biobank-uk and (www.ukbiobank.ac.uk/learn-more-about-uk-biobank/about-us/ethics). No patients were specifically involved in setting the research question or the outcome measures, nor were they involved in developing plans for recruitment, design, or implementation of this study. No patients were asked to advise on interpretation or writing up of results. There are no specific plans to disseminate the results of the research to study participants, but the UK Biobank disseminates key findings from projects on its website.

## SUPPLEMENTARY MATERIALS

Supplementary material for this article is available at http://advances.sciencemag.org/cgi/content/full/7/31/eabf8257/DC1
